# Aleutian Mink Disease Virus in Free-Ranging Mink from Sweden

**DOI:** 10.1371/journal.pone.0122194

**Published:** 2015-03-30

**Authors:** Sara Persson, Trine H. Jensen, Anne-Lie Blomström, Mia Tjernström Appelberg, Ulf Magnusson

**Affiliations:** 1 Division of Reproduction, Department of Clinical Sciences, Swedish University of Agricultural Sciences, Uppsala, Sweden; 2 National Veterinary Institute, Technical University of Denmark, Aarhus, Denmark; 3 Department of Biomedical Sciences and Veterinary Public Health, Swedish University of Agricultural Sciences, Uppsala, Sweden; University of Liverpool, UNITED KINGDOM

## Abstract

Aleutian mink disease (AMD) is a chronic viral disease in farmed mink and the virus (AMDV) has been found in many free-ranging mink (*Neovison vison*) populations in Europe and North America. In this study, AMDV DNA and AMDV antibodies were analysed in 144 free-ranging mink hunted in Sweden. Associations between being AMDV infected (defined as positive for both viral DNA and antibodies) and the weight of the spleen, liver, kidneys, adrenal glands and body condition were calculated and the sequences of ten AMDV isolates were analysed in order to characterize the genetic relationships. In total, 46.1% of the mink were positive for AMDV antibodies and 57.6% were positive for AMDV DNA. Twenty-two percent of the mink tested on both tests (*n* = 133) had dissimilar results. The risk of having AMDV antibodies or being positive for AMDV DNA clearly increased with age and the majority of the mink that were two years or older were infected. Few macroscopic changes were found upon necropsy. However, the relative weight of the spleen was sexually dimorphic and was found to be slightly, but significantly (*p* = 0.006), heavier in AMDV infected male mink than uninfected. No association between AMDV infection and body condition, weight of the kidneys, liver or adrenal glands were found. Several different strains of AMDV were found across the country. Two of the AMDV sequences from the very north of Sweden did not group with any of the previously described groups of strains. In summary, AMDV seems to be prevalent in wild mink in Sweden and may subtly influence the weight of the spleen.

## Introduction

Aleutian mink disease (AMD) in the American mink (*Neovison vison*) is caused by an amdovirus with high genetic diversity [1]. The AMD virus (AMDV) genome codes for two structural proteins (VP1 and VP2) and three non-structural proteins (NS1, NS2 and NS3) [2, 3]. There are several strains of AMDV, ranging from low virulent to highly virulent, thus, the disease will manifest differently depending on type of virus strain and host genotype [4, 5]. There are three general courses of infection in adult mink: persistent progressive AMD (resulting in death); persistent non-progressive; and transient with presumed elimination of the virus [4, 6, 7].

In adult mink, AMDV generally cause a chronic immune-mediated disease, characterized by high levels of antibodies with limited ability to eliminate the virus, followed by immune complex formation and deposition [7]. Early clinical signs in farmed mink are inappetence and weight loss, later followed by inactiveness and roughened coat occasionally with sprinklers/white hairs [8]. Other signs are infertility, small litters and abortions [9]. Mink with AMD may appear clinically healthy but often have poor reproduction [10]. Clinical signs in late stage disease are weakness, polydipsia, anaemia, diarrhoea, and incoordination of gait [8]. Macroscopic lesions seen upon necropsy of mink that have died of AMD can be emaciation, enlarged lymph nodes, splenomegaly, hepatomegaly and small shrunken kidneys with an irregular surface [6]. Early in the disease, the kidneys are enlarged and have petechial haemorrhage seen on the surface [6]. The length of the incubation period and the duration and course of the disease varies [8]. After infection, farmed mink can survive for a month, several months or up to several years, often depending on the genotype of the mink [6]. The virus can infect and spread between mink orally, by aerosol [11] and transplacentally [12].

Aleutian disease is common in Swedish mink farms, as no national stamping-out eradication program has been carried out. There are, however, some uninfected farms. Disease control is most commonly enforced by systematic culling of mink with high gammaglobulin titres (A-M Andersson, personal communication). During 2006 to 2009, the number of mink farms in Sweden decreased from 123 to 80 and the vast majority of the remaining farms were located in the south part of Sweden; only three were located in the middle of Sweden and none were located in the north part of the country [13]. Between 2006 and 2009, there have been at least 20 deliberate releases of farmed mink by Swedish animal rights activists [13]. Therefore, it is likely that AMDV has been transferred from farmed mink to wild mink. In addition, there are indications that AMDV is spilled back and forth between wild mink to farmed mink in Canada [14]. Antibodies and/or virus have been found in free-ranging American mink populations in Spain [15], England [16, 17], France [18], Canada [19, 20] and Denmark [21]. AMDV can also infect other species. In France, antibodies were found in European mink (*Mustela lutreola*), polecats (*Mustela putorius*), stone martens (*Martes foina*), pine martens (*Martes martes*) and common genets (*Genetta genetta*) [18]. Viral DNA has been found in otter (*Lutra lutra*) in Spain [15], and recently, a large screening for AMDV in different furbearing species in Canada found AMDV in six out of twelve species [22]. In experimental studies it seems that other species are not as severely affected by AMDV as mink, and some species could be potential reservoirs [23–25]. However, how wild populations of mink and other mustelids are affected by AMD is largely unknown.

The aim of this study was to describe the presence of AMDV in free-ranging mink from Sweden. Considering the observed manifestations in farmed animals, we hypothesized that free-ranging mink positive for virus and antibodies would be associated with larger spleen and liver, and also smaller or larger kidneys. Also, we hypothesized that being AMDV-infected would be associated with the amount of stored subcutaneous body fat (body condition), as a presumably persistent infection could affect the body condition of the wild mink negatively. The weight of the adrenals was also studied, as it could reveal a possible relation to stress and chronic disease [26, 27]. In addition, we determined the sequences of ten AMDV isolates from different parts of the country in order to characterize the genetic relationships of Swedish AMDV viruses.

## Material and Methods

### Sampling and sample preparation

**Fig 1:**
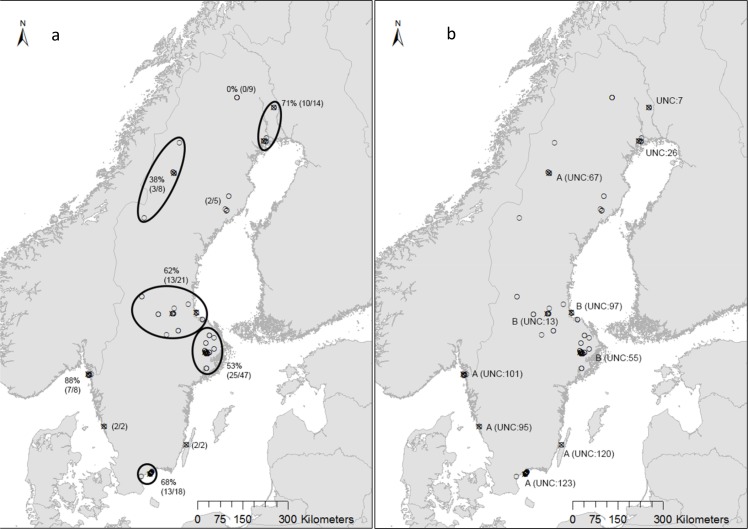
Free-ranging mink sampled for AMD antibody and AMDV virus detection (small circles) and virus sequence analysis (crosses). 1a) Percentage positive mink (by PCR)/total mink within each area (areas within ellipses are calculated together). 1b) Crosses are labelled with UNC number and phylogenetic group.

In total, 144 free-ranging mink carcasses were collected from mink hunters during 2004 to 2009. All mink were killed for nature protection measures during regular hunting activities and not driven by the study itself; therefore an ethical approval was not needed under Swedish legislation. The carcasses were frozen and transported to the necropsy facility where they remained frozen at -20°C until necropsy was performed. Of the 144 mink, 21 were caught in Listerlandet, a peninsula in Blekinge county in the southern part of Sweden (labelled “A UNC:123” in Fig 1), that has a high density of mink farms. This area is approximately 130 km^2^ and between 2006 and 2009 there were 50–60 mink farms located there. Age was determined by cementum analysis by Matson’s laboratory (Milltown, Montana, USA) [28], however, the mink from the area with high farm density were not included in this analysis. Assuming the mink were born the 1st of May, they were categorized into three age groups; juvenile (3–12 months old, n = 56), one year old (13–24 months, n = 48) and two or more years old (older than 24 months n = 18). According to hours of day length on capture date and site, the mink were divided into seasonal groups (spring, summer, autumn and winter), as previously described [29]. Upon necropsy, body weight was recorded and the mink were inspected for gross lesions and the internal organs were dissected and weighed. The subcutaneous fat pat situated between the hind legs on the ventral part of the abdomen were dissected and weighed. Body condition was set as subcutaneous fat (g)/body mass (kg). Blood was collected by pipette from the heart and/or *vena cava caudalis* and refrozen at -70°C. The spleen was refrozen (-20°C) until further analysis. Blood from 142 mink were thawed and transferred in duplicates to sampling paper according to instructions (Fin Furlab Oy, Vaasa Finland).

### Analysis of AMDV/AMDV antibodies and sequence analysis

Specific AMDV antibodies were analysed using enzyme-linked immunosorbent assay (ELISA), with a sensitivity of 99% and a specificity of 97% [30, 31]. DNA, used as template in the AMDV PCR, was extracted from spleens from 139 of the collected mink using by QIAamp Blood Mini kit according to manufacturer’s instructions (Qiagen, Hilden, Germany). A fragment of 374 bp of the NS1 gene was amplified by PCR as described previously [32].

To perform genetic characterization and phylogenetic analysis of AMDV circulating in the free-ranging mink population in Sweden 10 of the positive PCR products were sent for sequencing (marked with crosses in Fig 1). Sequencing was performed with the PCR primers by LGC Genomics, Berlin, Germany [32]. The sequences were assembled and edited using SeqMan (Lasergene 9, DNAStar). Seventy-four AMDV NS1 nucleotide sequences were downloaded from the GenBank database including sequences from the main 4 groups (A-D) previously identified by Olofsson *et al* [1]. ClustalW alignment was performed using MEGA5 [33] as were the phylogenetic analysis. The phylogenetic tree was constructed using the Neighbour-joining algorithm with p-distance and a bootstrap value of 1000. Sequence identity plot was performed using the Bioedit software (http://www.mbio.ncsu.edu/bioedit/bioedit.html). The sequences from this study have been submitted to GenBank and are available under the follow accession numbers: KC790413.1 (UNC-123); KC790412.1 (UNC-120); KC790411.1 (UNC-101); KC790410.1 (UNC-97); KC790409.1 (UNC-95); KC790408.1 (UNC-67); KC790407.1 (UNC-55); KC790406.1 (UNC-26); KC790405.1 (UNC-13); KC790404.1 (UNC-7).

### Statistical analysis

The Glimmix procedure of SAS (SAS Institute Inc., Cary, NC, USA, version 9.02.01) for logistic regression was used to test the effect of age class on the ELISA and PCR results from the wild mink. Sex and sampling season were also tested in this model but none were included due to insignificance. Odds ratios (OR) were obtained by this procedure. In order to characterize the impact of AMDV infection on free-ranging mink populations, mink that were positive for both AMDV-specific antibodies and viral DNA (n = 49) were considered having an ongoing AMDV-infection with an activated immune response. These mink were included in a binary variable (AMDI) together with mink that were negative on both tests and therefore considered unaffected (n = 48). For examining the influence of this variable on the body condition and the relative spleen, liver, kidney and adrenal weight (% of body mass), the general linear model (GLM) procedure of SAS was used. As organ weights and body condition may be influenced by other factors such as sampling season, age, sex and the weight of the subcutaneous fat [29], these factors were also tested and included in the models when significant. When needed, the dependent data were log-transformed to improve normality of the residuals. P-values less than 0.05 were considered significant. As there was a significant effect of sex on relative spleen and kidney weights, the effect of the variable AMDI was calculated for females (n = 24) and males (n = 73) separately. As there could be a quite high probability that some of the mink caught in the area with high farm density were recent fur farm escapees, those mink were excluded from the regression models and are not included in the term “free-ranging mink” throughout the manuscript.

## Results

At necropsy six mink that were seropositive and/or PCR positive for AMDV had lesions which could, in theory, be related to AMD. Three mink had granulated kidneys and one had visibly enlarged kidneys. There were two mink with spleen lesions; one with swollen spleen and one with protruding nodules on cut surface. All other mink appeared to be healthy and parasites were rare. Of the mink caught in the area with high farm density, two mink had coat colours usually only seen on mink bred in farms; dark black and grey, indicating that they could be escapees from mink farms. These two mink had a lot of subcutaneous fat and one of them had enlarged mesenteric lymph node and the spleen appeared to be swollen. Both mink were seropositive and viral DNA was detected in the spleens.

The number of animals tested positive or negative for AMDV antibodies and AMDV DNA are shown in Table 1. Sixteen of the 23 mink that were positive by PCR but had no detectable antibodies by ELISA were sent for age determination, and the result was 12 juveniles and 4 one-year-olds. Fifty-one percent of 45 females and 57.7% of 97 males were positive for AMDV DNA. When excluding the mink from the area with high farm density, 47.1% (of 123) were seropositive and 51.2% (of 121) were positive for AMDV DNA. In four mink the ELISA result was inconclusive, i.e. there was one positive and one negative result for each mink (these were excluded from statistical calculations on the ELISA results).

**Table 1:** Percentage of positive and negative mink for detection of AMDV antibodies (ELISA) or AMDV DNA (PCR).

	Percentage	Number of mink/total
ELISA +	46.1%	65/142
PCR +	57.6%	80/139
		
ELISA+/PCR+	40.6%	54/133
ELISA-/PCR-	37.6%	50/133
ELISA+/PCR -	17.3%	23/133
ELISA-/PCR+	4.5%	6/133

**Fig 2:**
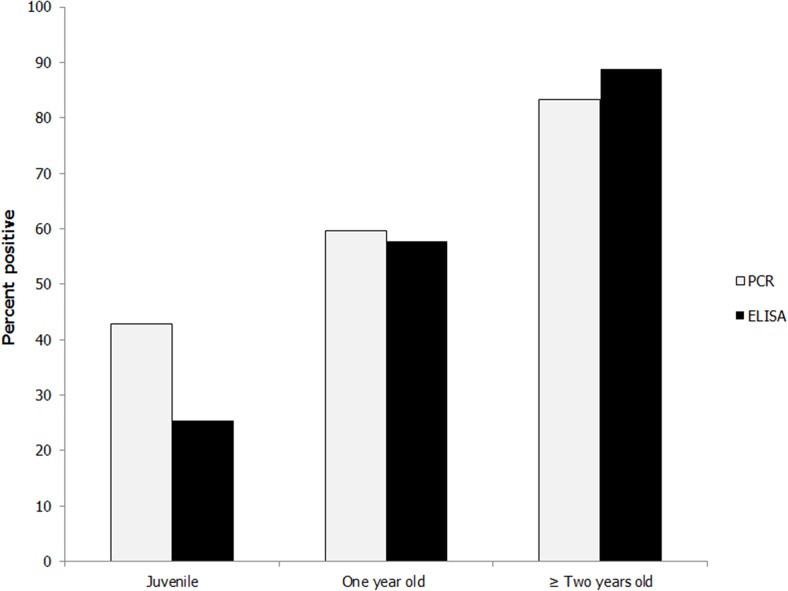
Percent mink positive for viral DNA (PCR) and AMDV antibodies (ELISA) in three different age groups; juvenile (24 of 56 mink and 14 of 55 mink, respectively); one year old (28 of 47 mink and 26 of 45 mink, respectively) and mink two years or older (15 of 18 mink and 16 of 18 mink, respectively).

In the logistic regression model (Table 2), the age class significantly influenced the outcome of both ELISA and PCR tests. The percent positive for each age class is shown in Fig 2. Compared to juvenile mink, the odds of being positive on the ELISA test were 3.7 times higher for one year old mink and 22 times higher for mink that were two years or older. The same comparison on the PCR results gave an OR of 2.0 for one year old mink, and for mink two years or older an OR of 6.7 for being positive. There were 10 mink that were two years old, 7 mink that were three years old and one five-year-old.

**Table 2:** Odds ratio estimates and effect of age class from the logistic regression model.

Response variable	Effect	*n*	P	OR	95% confidence interval
AMDV antibodies	Age^a^	119	0.0001		
	≥2 years old	18		22	4.4–108
	1 year old	45		3.7	1.6–8.7
	juvenile	56			
AMDV DNA	Age^a^	121	0.02		
	≥2 years old	18		6.7	0.9–4.4
	1 year old	47		2.0	1.7–26
	juvenile	56			

a Type III test of fixed effect

The mean relative spleen weight in males (2.7 g/kg BW), was significantly lower (p = 0.002) compared with that of females (3.3 g/kg BW). The mean absolute spleen weight was 2.9 g for males and 1.9 g for females. In male mink, there was a significant difference (*p* = 0.006, R^2^ = 10%) in relative spleen weight between mink that were positive for AMDV infection (2.9 g/kg BW) and mink that were negative for AMDV infection (2.4 g/kg BW). No significant difference was found among female mink. AMDV infection was not associated with relative kidney, liver or adrenal weight, nor did it influence the body condition of the mink. There were, however, a tendency (*p* = 0.08) for a difference in relative kidney weight between male mink that were positive for AMDV infection (8.7 g/kg BW) and male mink that were not infected (8.4 g/kg BW).

**Fig 3:**
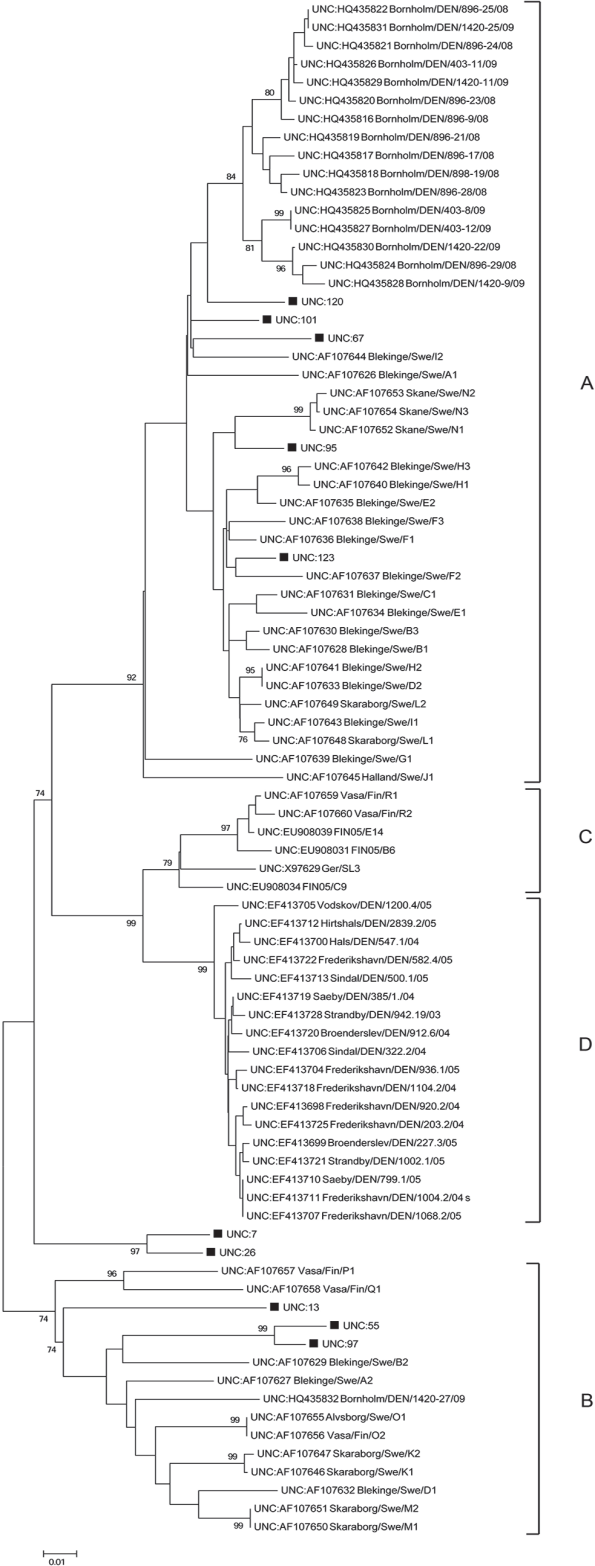
The neighbour-joining tree displays the phylogenetic relationship between NS1 sequences of AMDV from wild mink in Sweden and 74 NS1 AMDV sequences available from GenBank. Groups (A-D) established by Olofsson et al [1] are indicated in the tree. The NS1 AMDV sequences from this study are marked with ■.

The sequence analysis showed a rather high sequence variation in the analysed NS1 region. On nucleotide level, the sequences from this study showed a 97.5–81.5% nucleotide sequence identity to each other, slightly lower values were seen on protein level (94.6–70.9% sequence identity). The phylogenetic analysis (Fig 3) showed that five (50%) of the sequences belonged to group A, three to group B and two of the 10 sequences grouped in a separate clade. There is a certain degree of geographical clustering of the NS1 sequences from the free-ranging mink in this study (Fig 1). All the three sequences belonging to group B came from the middle of Sweden, while the two sequences that grouped separately from other AMDV NS1 both came from the northeast of Sweden. For group A, all the sequences came from mink sampled in the southern areas of Sweden, with the exception of one sample that was from the northern parts of Sweden. To verify the phylogenetic relationship displayed using the Neighbour-joining method, tests like maximum-likelihood and minimum-evolution were also conducted on the same datasets, and all showed similar results (data not shown).

## Discussion

At present, little data has been available on the presence of AMDV in free-ranging mink in Sweden [13]. Therefore, this study aimed to perform a screening and partial genetic NS1 characterization of AMDV in free-ranging mink in Sweden and investigate any manifestation associated with infection upon macroscopically performed necropsy. There were few necropsy findings in AMDV positive mink that could be associated with AMD, and most mink appeared healthy.

The virus seemed to be spread all over the country, with slightly lower percentage of positive mink in the north-northwest, although this observation has to be interpreted with caution as the samples were not evenly distributed across Sweden. Even so, the northern part of the country has historically had relatively few mink farms [34] and at the time of sampling, no mink farms at all. Notably, in Canada it seems that seroprevalence is higher in areas close to mink farms [20]. In total, 58% of all the mink in the current study were positive for Aleutian disease virus DNA. The highest reported proportion of AMDV positive mink is from Nova Scotia, Canada, where 56 of 60 (93.3%) of wild mink were PCR positive [22], which is much higher than the previously reported 25% (of 183) in mink from different parts of Canada [14]. The percentage of seropositive mink in the present study (46%), were similar or higher in comparison to what has been reported in free-ranging mink in Europe. In France, 23% (of 75) were seropositive [18] and similar results were seen in Canada (29% (of 208)) [20]. In mainland Denmark, only 3% (of 365) were seropositive, while 45% of 142 mink from the Danish island Bornholm were found to have AMDV antibodies [21].

The proportion of mink positive for AMDV increased with age. For mink that were 2 years or older, the odds of being seropositive were 21.9 times as large as for juvenile mink, which means that after two year of age almost all mink were infected with AMDV (see Fig 2). This result underlines that AMDV infections are probably very common in the wild Swedish mink population. There were six mink with antibodies but no detectable AMDV DNA in their spleen which indicate that these mink could possibly have cleared the virus. Another, and perhaps a more plausible explanation could be that the virus, even though often detected in spleen [35], were present in other organs, for example lymph nodes [36].

Some mink had virus in their spleen, but no antibodies could be detected. A possible explanation is that either the PCR or ELISA could have generated false positive or false negative results. Some, but not all, of these samples were taken from mink with more advanced decomposition, which could have hampered the analysis as decomposition may cause DNA degradation. It is also possible that these mink were newly infected, and had not produced detectable amounts of antibodies yet. It has been seen in an experimental study that it can take up to 4–7 weeks before antibodies are detected after intranasal inoculation [5]. In our study, the majority of the antibody negative but virus positive animals were juveniles, supporting the theory that they were newly infected mink.

A difference in relative spleen weight between the sexes was found in this study. This has been reported earlier [37] and the reason for the difference is not clear. In the male mink in this study, the relative spleen weight was associated with being AMDV positive and having AMDV antibodies, but not in the female mink (possibly due to a smaller sample size). Enlarged spleen is the most common macroscopical finding upon necropsy of farmed mink with subclinical AMD [10]. Therefore, it is recommendable to take AMDV infection status into account when assessing spleen weights from wild mink. However, the difference between the mean relative spleen weights was very small. It should be emphasised that enlargement of the spleen is an unspecific finding. It has, for example, been seen in outbreaks of toxoplasmosis in farmed mink [38] and could possibly be an effect of parasite load [39].

Until now, no sequencing of AMDV in free-ranging mink has been performed in Sweden and thus only sequence data from farmed mink in the southern part of the country has been available. Olofsson *et al*. showed a high genetic diversity of AMDV in farmed mink in Sweden and identified three different phylogenetic groups (A-C) [1]. Yet another phylogenetic group (D) has been proposed by Christensen *et al*. [40]. In the current study, we show that at least two (A and B) of the four genetic AMDV groups that have been proposed previously in farmed mink circulate in free-ranging mink in Sweden. Two of the sequences from the very north of Sweden, with a sequence difference of 24–12% to the other sequence used in the phylogenetic study, did not group with any of the known groups (A-D). To verify that these strains constitutes a new separate group further sequence analysis is needed—sequencing longer portions of the gene as well as other genes. The nucleotide sequence identities between the NS1 AMDV sequences from this study were 97.5–81.5% and thus the degree of variation to each other and to those from other studies correspond well to the observed variation seen in other AMDV studies [1, 21].

## Conclusions

This study shows that AMDV seems to be endemic all over Sweden and that the percentage of free-ranging mink that are AMDV positive and/or have AMDV antibodies appears to be high, with several different strains circulating across the country. It seems that similar AMDV strains exist in farmed and free-ranging mink in Sweden. The risk of a mink being AMDV infected clearly increased with age. Although many mink were positive for AMDV, most mink appeared healthy and only little evidence of clinical disease were found upon macroscopic investigation. More detailed studies, including for example histopathology, are needed in order to understand the impact of this disease on the health of free-ranging mink.
